# Life as a guide to prebiotic nucleotide synthesis

**DOI:** 10.1038/s41467-018-07220-y

**Published:** 2018-12-12

**Authors:** Stuart A. Harrison, Nick Lane

**Affiliations:** Centre for Life’s Origin and Evolution, Department of Genetics, Evolution and Environment, University College London, London, UK

## Abstract

Synthesis of activated nucleotides has been accomplished under ‘prebiotically plausible’ conditions, but bears little resemblance to the chemistry of life as we know it. Here we argue that life is an indispensable guide to its own origins.

## Introduction

“The justification of prebiotic syntheses by appealing to their similarity to enzymatic mechanisms has been routine in the literature of prebiotic chemistry. Acceptance of the RNA World hypothesis invalidates this type of argument. If the RNA World originated de novo on the primitive Earth, it erects an almost opaque barrier between biochemistry and prebiotic chemistry.” Leslie Orgel 2004.

There is little doubt that RNA is central to the origins of genetic information and protein synthesis, but that is a far cry from the stronger statement, articulated by Orgel, that if metabolism arose from an RNA world, then life can give no insights into its own origin^1^. Yet this view resonates with much prebiotic chemistry over the last decade. Activated pyrimidine and oxopurine nucleotides have been synthesised at good yield from purportedly prebiotically plausible precursors such as cyanide and cyanoacetylene, driven by UV radiation^2^. Wet-dry cycles in the presence of laminated clay minerals and lipids can polymerise nucleotides to RNA^3^. Accordingly, some claim that the problem has already been solved, at least conceptually.

Has it? Not in everybody’s view. Perhaps the biggest problem is that the chemistry involved in these clever syntheses does not narrow the gap between prebiotic chemistry and biochemistry—it does not resemble extant biochemistry in terms of substrates, reaction pathways, catalysts or energy coupling^4^. Does that matter? Those with chemical acuity claim to see ‘strategic similarities’ to biochemistry^5^, but biochemists are apt to disagree^6^.

**Fig. 1:**
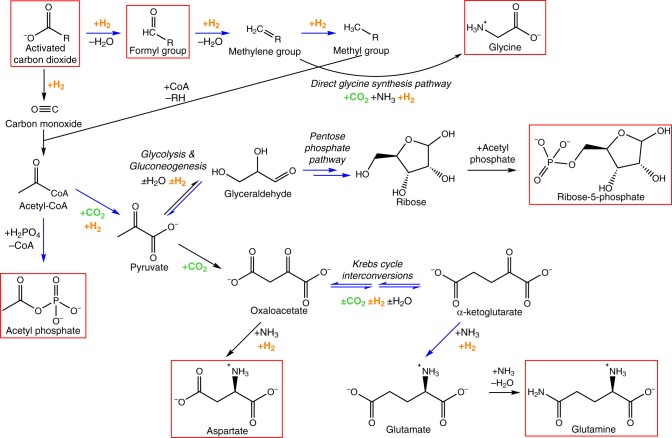
Schematic of what could have been the beginnings of biochemistry. Main steps of core intermediary metabolism starting from CO_2_ and H_2_, showing key intermediates in the acetyl CoA pathway, reverse incomplete Krebs cycle, reductive amination, gluconeogenesis and the pentose phosphate pathway. The precursors for nucleotide synthesis are boxed in red. Reactions shown in blue have been partially or completely achieved under prebiotic conditions. R denotes biological cofactors such as tetrahydrofolate; prebiotic equivalents could include mineral surfaces. Acetyl phosphate can phosphorylate ADP by substrate-level phosphorylation in bacteria and archaea, and under prebiotic conditions. Ribose phosphate is the precursor for phosphoribosyl pyrophosphate, used in all nucleotide synthesis. Importantly, CO_2_ and H_2_ equivalents are required at multiple steps

In the broadest terms, life hydrogenates carbon dioxide, using ion gradients across membranes to lower the kinetic barriers^7–9^. The deepest branches in the tree of life are autotrophic and chemiosmotic^9^. Just six pathways of CO_2_ fixation are known; the most ancient generate carboxylic acids, notably Krebs cycle intermediates^7,10,11^. Carboxylic acids are at the centre of intermediary biochemistry across all life (Fig. 1): amino acids, sugars and fatty acids are formed from Krebs cycle intermediates; nucleotides are made from amino acids and sugar phosphates^10,11^. The difficult condensation reactions to form nucleotides and polymers including RNA, DNA and polypeptides are accomplished in water, using ATP. None of this bears any resemblance to cyanosulphidic protometabolism or wet-dry cycles in UV-seared volcanic pools.

Can life be used as a guide? Phylogenetic reconstructions of the tree of life are fraught with difficulties but tend to depict the last universal common ancestor of life, LUCA, as a CO_2_-dependent autotroph^12^. The difficulty is extrapolating backwards from LUCA to prebiotic chemistry. LUCA certainly had genes and proteins, and that level of complexity is undeniably a long way from prebiotic chemistry. But that does not mean that life can offer no insights into the environment in which it began. de Duve argued that the only scientifically plausible explanation for the appearance of biological catalysts, whether enzymes or ribozymes, is selection^13^. Enzymes can only be selected if they amplify processes that occur spontaneously, driven by natural disequilibria and catalysed by inorganic ions, mineral catalysts or small organics. Selection therefore imposes a link between geochemical flux and metabolism, which implies continuity between the biochemistry of LUCA and protometabolic flux in a specific environment^13^. This argument for true (not strategic) similarity is reinforced by what has been called the end-product problem^6^. Metabolic pathways generate end-products that are ultimately selected because they are useful, but not all intermediates along the pathway are obviously useful, except as a step along the way. The idea that metabolic pathways evolved stepwise from the beginning (Granick hypothesis) or the end (retrograde hypothesis) is problematic in that it implies every intermediate has a use, or that each one accumulates in the environment, respectively. Neither matches the reality of fleeting, often unstable intermediates that do not accumulate precisely because they are reactive^6^.

This selectionist view predicts that entire prebiotic pathways, mirroring extant metabolism, could exist under propitious conditions. That idea was vividly dismissed by Orgel as “if pigs could fly” hypothetical chemistry^14^, but recent research shows that intermediates along whole metabolic pathways, including the acetyl CoA pathway and Krebs cycle, really can form interchangeably at low concentrations^6,15,16^. In this respect, the synthetic chemists’ historical preoccupation with generating high yields of specific products is a red herring. If selection acts by increasing the specificity and yield of end-products, perhaps by enhancing catalysis of the slowest or least likely steps of a spontaneously occurring pathway, then prebiotic chemists should be aiming to detect not high yields of specific end-products, but rather low concentrations of various intermediates along the core biochemical highways.

Could prebiotic nucleotide synthesis follow a similar pattern? It is, admittedly, a tall order—in bacteria and archaea, 12 enzymatic steps are required for the synthesis of purine nucleotides. But there are still patterns that have not been considered seriously enough by prebiotic chemists. Nucleotide synthesis requires phosophoribosyl pyrophosphate, amino acids (aspartate, glutamine and glycine) and C1 formylations or carboxylations. The ease with which purines such as adenine can be synthesised from cyanide meant that prebiotic chemists long tried (and failed) to react adenine with ribose to form adenosine. But that is not what life does. Purine bases are built sequentially onto ribose-5-phosphate, which acts like a handle, with a common binding motif in most of these enzymes. That implies the importance of surface catalysis. The apparent paradox that ATP is required for six condensation steps in purine synthesis is offset by the possibility that simpler prebiotic phosphorylating agents such as acetyl phosphate could potentially drive these steps. Acetyl phosphate is the fulcrum between thioester and phosphate metabolism; it can form spontaneously by phosphorolysis of thioacetate under neutral to alkaline (pH 7–9) and ambient to warm (20–60 °C) conditions^4^. Once formed, it can phosphorylate ribose and even ADP directly under equivalent conditions, narrowing the gap between geochemistry and the origins of intermediary metabolism^4^.

Attempting to emulate life by using amino acids, sugar phosphates and ATP equivalents to synthesise nucleotides might seem pointless when prebiotic chemists have already produced them using simpler, more reactive compounds such as cyanide as the starting point. But if we want to understand how life started on Earth, and possibly elsewhere, we need to understand why life does not, in fact, work that way. If life began as it was to continue, by hydrogenating carbon dioxide, then we need to investigate environments in which geochemical flux could drive analogous protometabolic pathways, giving rise to the synthesis, growth, self-organization, and hereditary replication of matter^17^.

Alkaline hydrothermal vents present tantalising parallels to cells^9^. In the Hadean, these vents should have been saturated in H_2_ and CO_2_, the feedstock for growth via the acetyl-CoA pathway and reductive Krebs cycle^7–12^. Their microporous structure should have facilitated vectorial proton chemistry similar to that used for CO_2_ reduction in extant cells—the pH range in vents (~5–11) should form natural proton gradients across thin barriers that modulate the reduction potential of H_2_ and CO_2_, lowering the kinetic barrier to their reaction, as in modern cells^7^. The thin barriers should have contained transition metals, nickel and iron in particular, found in key enzymes in ancient metabolic processes such as ferredoxin^8^. The temperature range (50–90 °C) is theoretically conducive to the synthesis of amino acids, fatty acids and sugars^10^. Indeed, when CO_2_ is the electron acceptor under hydrothermal conditions, carboxylic acids are formed, as in life. With iron as an electron donor and metal ions as catalysts, most intermediates in the acetyl CoA pathway and reverse Krebs cycle have been produced^6,15,16^. Long-chain fatty acids and fatty alcohols have been synthesised under hydrothermal conditions (150 °C), albeit so far only in steel reactors, suggesting that iron might be a critical electron donor or catalyst^18^. Amino acids are produced by reductive amination of α-keto acids such as pyruvate and α-ketoglutarate under alkaline conditions (pH 9–11, 50–75 °C, FeS catalysts)^19^. Sugars, including ribose, are made from formaldehyde under alkaline hydrothermal conditions (pH 11, 60 °C) via the formose reaction^20^.

Equivalent reductions of CO_2_ using H_2_ rather than native iron as the main electron donor have not yet been reported, but microfluidic experiments that imitate life’s vectorial chemistry are in their infancy. Hydrogen gas is difficult to work with in high-pressure through-flow reactors so little serious work has been done. But plentiful hydrogen is needed to drive protometabolism forwards (Fig. 1). Because most steps of most metabolic pathways are close to equilibrium, reactions can proceed in either direction. Net growth is achieved not because individual steps are irreversible but because the environment is far from equilibrium. In alkaline hydrothermal vents, the high partial pressure of H_2_ thermodynamically favours CO_2_ reduction, driving biosynthesis and growth rather than catabolic breakdown of organics back to H_2_ and CO_2_. In other words, hydrothermal flux drives growth.

What should we conclude? Alkaline hydrothermal pores resemble autotrophic cells in their structure, chemistry and mineral composition. Simulations under hydrothermal conditions form the products of intermediary biochemistry required for nucleotide synthesis. We are therefore attempting to synthesize nucleotides under equivalent conditions. Back in 1953, Miller showed that amino acids could be formed through electrical discharges on reduced gases. Few researchers still believe that this is how amino acids were actually formed at the origin of life, and it now seems almost embarrassingly easy to make amino acids under wide-ranging conditions. The demonstration that activated nucleotides can be formed from cyanide is a finding of comparable significance to the Miller-Urey experiment—it proves it can be done, and it eliminates some of the mystique. But it does not prove this is the only way to do it. Life itself hints that this was not the way it happened. If we want to understand the origin of life, we would be foolish to ignore life as a guide.
